# Implantable Direct Current Neural Modulation: Theory, Feasibility, and Efficacy

**DOI:** 10.3389/fnins.2019.00379

**Published:** 2019-04-18

**Authors:** Felix P. Aplin, Gene Y. Fridman

**Affiliations:** ^1^Department of Otolaryngology Head and Neck Surgery, Johns Hopkins University, Baltimore, MD, United States; ^2^Department of Biomedical Engineering, Johns Hopkins University, Baltimore, MD, United States; ^3^Department of Electrical and Computer Engineering, Johns Hopkins University, Baltimore, MD, United States

**Keywords:** direct current, neuromodulation, neural implant, electrical stimulation, neural interface, tDCS, neural block, synaptic remodeling

## Abstract

Implantable neuroprostheses such as cochlear implants, deep brain stimulators, spinal cord stimulators, and retinal implants use charge-balanced alternating current (AC) pulses to recover delivered charge and thus mitigate toxicity from electrochemical reactions occurring at the metal-tissue interface. At low pulse rates, these short duration pulses have the effect of evoking spikes in neural tissue in a phase-locked fashion. When the therapeutic goal is to suppress neural activity, implants typically work indirectly by delivering excitation to populations of neurons that then inhibit the target neurons, or by delivering very high pulse rates that suffer from a number of undesirable side effects. Direct current (DC) neural modulation is an alternative methodology that can directly modulate extracellular membrane potential. This neuromodulation paradigm can excite or inhibit neurons in a graded fashion while maintaining their stochastic firing patterns. DC can also sensitize or desensitize neurons to input. When applied to a population of neurons, DC can modulate synaptic connectivity. Because DC delivered to metal electrodes inherently violates safe charge injection criteria, its use has not been explored for practical applicability of DC-based neural implants. Recently, several new technologies and strategies have been proposed that address this safety criteria and deliver ionic-based direct current (iDC). This, along with the increased understanding of the mechanisms behind the transcutaneous DC-based modulation of neural targets, has caused a resurgence of interest in the interaction between iDC and neural tissue both in the central and the peripheral nervous system. In this review we assess the feasibility of *in-vivo* iDC delivery as a form of neural modulation. We present the current understanding of DC/neural interaction. We explore the different design methodologies and technologies that attempt to safely deliver iDC to neural tissue and assess the scope of application for direct current modulation as a form of neuroprosthetic treatment in disease. Finally, we examine the safety implications of long duration iDC delivery. We conclude that DC-based neural implants are a promising new modulation technology that could benefit from further chronic safety assessments and a better understanding of the basic biological and biophysical mechanisms that underpin DC-mediated neural modulation.

## Introduction

One of the earliest direct interactions with nervous system was conducted by Luigi Galvani in the late 1700s. In Galvani's experiments, isolated frog leg muscles, and the incoming nerves were depolarized with impulse-like delivery of electrical current. Galvani was confined to using a statically charged rod or metal rods attached with chains to Leyden jar capacitors to evoke a muscular response (Geddes and Hoff, 1971; Piccolino, 1998). Once Alessandro Volta developed a battery in the early 1800's, he tested the effects of delivering ~50 V of direct current (DC) to neurons by attaching the leads to various parts of his own body, including the ear, eye, and tongue. His famous observations of the sensations are well-known in the field of neuromodulation, with descriptions of crackling, pain, noise, and shocks. While the mechanism of electricity-to-nervous system interaction was not well-understood at the time, one interpretation of these early experiments is that both electrical pulses and direct current can both be used to successfully interact with the nervous system (Guleyupoglu et al., 2013).

The first fully implantable pacemaker was designed and implanted in 1958 by Rune Elmqvist and Åke Senning, at the Karolinska Hospital in Sweden (Aquilina, 2006). This device delivered regularly spaced electrical current to the heart. A 1.5 ms pulse initiated a heart compression, but continued heart activity was self-propagating for another 1 s with no output from the pulse generator. These electrical pulses were delivered to a metal electrode implanted in the heart muscle. The need to deliver a very short duration pulse to depolarize the cardiac tissue of the heart fortuitously coincided with the fact that one could not deliver longer duration electrical current to a metal electrode without creating potentially toxic electrochemical byproducts. The inventors were not necessarily considering this implication during the development process, but the device was safe largely because the pulses were short with long inter-pulse intervals.

The pacemaker was self-contained and battery powered, giving the patient the freedom to move around. In modern terminology, the pacemaker was the first implantable pulse generator (IPG). The invention of this IPG created a precedent that showed us how we could effectively evoke electrical activity in the body safely with a device that delivered short pulses. In the nervous system, pulsatile stimulation was in principle confined to evoking an action potential (AP) in response to a pulse. However, due to the gross spatiotemporal spike rate coding method of the peripheral nervous system, pulsatile stimulation offered such a broad realm of applications that IPG technology dominated the field of neuromodulation for many years and it is still the primary commercial technology for neuromodulation therapies (Loeb, 2018).

DC was nearly abandoned for the purposes of implantable neural stimulation due to the therapeutic success and broad validation of IPGs in various applications, and the technical barrier associated with the challenge of safely delivering electrical current for a prolonged duration to a metal electrode implanted in biological tissue (Merrill et al., 2005). The recent resurgence of this mode of neuromodulation is due to technical innovations that have allowed DC to be delivered for longer durations to neural targets, and the success of experiments conducted through electrodes positioned on the skin where safety concerns could be more easily mitigated (Ruffini et al., 2013; Bikson et al., 2016). The other reason that DC neuromodulation has gained interest in recent years is that the field of neuromodulation has become refined sufficiently to be faced with new challenges that are more difficult to address using pulsatile waveforms. Because DC directly controls membrane potential, it can increase or decrease firing rate, altogether block neural activity, control AP propagation velocity, and modulate synaptic connectivity (Goldberg et al., 1984; Bikson et al., 2004; Vrabec et al., 2017; Strang et al., 2018; Yang et al., 2018). DC also appears to maintain the stochastic properties of AP inter-pulse intervals on each neuron (Goldberg et al., 1984), in contrast to conventional pulsatile stimulation for which an evoked AP in phase with the stimulation pulse is the intended effect.

This review covers the recent technological advances to deliver direct current to neurons, our understanding of how DC electric fields interact with neurons, potential therapeutic applications of DC, and finally the safety considerations associated with delivering DC to neural tissue.

## DC Enabling Technology

### The Tissue-Electrode Interface

The interface between an electronic neural implant and biological tissue is called an electrode. One side of the electrode surface is exposed to electrons, while the other is exposed to ions in the body fluids. If the electrons congregate temporarily on one side of this interface, on the other side they cause positive ions to move toward this interface and the negative ions to move away. If they are left there for a longer duration, some of these electrons will cross into the solution, causing a chemical reaction by breaking up or creating molecular bonds. On the opposing electrode, the opposite effect occurs, where lack of electrons will attract an electron from a negative ion in the solution. This general principle is one of the fundamental mechanisms behind electrochemistry (Zoski, 2007). Unless carefully and intentionally controlled, electrochemical reactions occurring at the metal-tissue interface are generally harmful to the body processes, causing pH changes, electrode corrosion with toxic byproducts, and bubble formation due to electrolysis (Brummer et al., 1983; Shannon, 1992; Merrill et al., 2005; Pour Aryan et al., 2014). For this reason, IPG designers are careful to avoid any unwanted electrochemical reactions when using bare metal electrodes to deliver current to the body. This can be accomplished in three ways: by decreasing the amount of time that the electrode is exposed to excess of electrons by reducing pulse duration; by limiting the amplitude of the current pulse that is delivered during this stimulation time and consequently the number of electrons that congregate at the electrode; and by increasing the surface area over which these electrons are distributed to reduce their density. IPGs typically use charge balanced biphasic pulses on the order of microseconds to milliseconds per phase to interact with neurons (Merrill et al., 2005; Pour Aryan et al., 2014). For the typical bare metal Pt electrodes, the safety criterion is 300 μC/cm^2^ (Shannon, 1992; Merrill et al., 2005; Pour Aryan et al., 2014). This type of safety criterion is referred to as the “charge injection criteria” and it is defined as the charge per electrode area necessary to cause electrolysis.

### Improving Charge Injection Criteria

Delivering direct current using a metal-tissue interface designed to function with an IPG is not possible without violating charge injection criteria and thus causing electrochemical reactions at the surface of the electrode. Development of electrode design has focused on improving charge injection limits, either as a means to decrease electrode size and thus improve the resolution of stimulation, or to deliver electrical current for very long durations in applications for which short pulses are not as effective, such neural block (Vrabec et al., 2016, 2017). The gradual evolution of these technologies has helped to enable the development of devices capable of safe DC delivery.

Improvements to the charge injection criteria necessary to enable a decrease in the electrode size have been addressed primarily with improvements in electrode surface treatments that increase the electrode area (Won et al., 2018). Another approach is to coat the electrodes with a dielectric oxide, such as that used for the Ta-Ta^2^O^5^ electrode, which can be used to prevent electrons from crossing the boundary into the solution without increasing the surface area (Brummer et al., 1983). This benefit comes at the cost of increased voltage needed to deliver the same amount of current, since the capacitance of the electrode drops in proportion to the thickness of the oxide. These treatments are primarily designed to maintain the delivery of charge for a smaller electrode size and they increase charge injection capacity by as much as 4 mC/cm^2^ (Guyton and Hambrecht, 1973, 1974).

When the design goal is to deliver current for durations that are much longer than those needed to evoke an action potential (to maintain a neural block for example) the modifications to the electrode design must accommodate several orders of magnitude increase in charge injection criteria. One improvement over the surface treatment method is the use of polymer coatings. This method creates a three-dimensional analog of the two-dimensional electrode surface. These coatings are collectively referred to as hydrogel polymer coatings, with PEDOT:PSS as the most well-known of these (Nyberg et al., 2002; Ferlauto et al., 2018). These polymers conduct electronic current and have the property of absorbing the surrounding electrolyte (e.g., body fluid) that allows for a capacitive interface between ions and electrons to form on a molecular scale throughout the polymer chains. Hydrogel polymer coatings have been able to achieve safe charge injection capacities as high as 34 mC/cm^2^ (Nyberg et al., 2002).

Although electrode surface coating can improve charge injection capacities by orders of magnitude, they also introduce new complications. The addition of another material interface can create additional toxicity concerns: for example, carbon nanotube coatings can improve charge injection and impedance properties of metallic electrodes but may also increase cytotoxicity and inflammation at the electrode interface, either due to intrinsic properties of the material (Shvedova et al., 2003; Gilmour et al., 2013) or via chemicals generated as a result of coating deposition (De Volder et al., 2013). Roughened rigid surfaces generate mechanical tissue stress, and along with hydrogel based surfaces are potentially more brittle and thus less stable for chronic implantation (Aregueta-Robles et al., 2014). While none of these limitations are hard barriers to implementation, they prevent a “one-size-fits-all” approach to continued improvement of charge injection capacity in neural implants.

### Ionic Direct Current Delivery

Further attempts to increase the duration of current delivery have come in two forms: creating a barrier between the body and the electrochemical byproducts at the electrode, and technology that eliminates or heavily mitigates toxic electrochemical reactions at the electrode while maintaining direct *ionic* current (iDC) flow. Both deliver DC to the tissue under the assumption that the electrochemical toxicity at the metal electrode is avoided for the duration of stimulation and the biological tissue is never exposed to the toxic chemical byproducts.

An electrochemical barrier can be accomplished by physically separating the electrode and the chemical corrosive byproducts with a column of electrolyte or electrolytic gel. One example of such an electrode is the Separated Interface Nerve Electrode (SINE). This concept introduced silicone tubing filled with an electrolyte between a syringe that contained a metal electrode and the target nerve (Ackermann et al., 2011; Vrabec et al., 2016, 2017). It is also possible to gel the electrolyte with Agar to mechanically stabilize it in the column and prevent the electrolyte from potentially leaking (Fridman and Della Santina, 2013a). Other methods involve complex chemical film coatings that “sequester” electrons in a chemical Faradaic reaction, whose products do not diffuse into the solution and are reversible. One example of such interface is activated iridium (Brummer et al., 1983; Beebe and Rose, 1988). In the case of activated iridium, the introduction or removal of an electron results in transitions between Ir^3+^ and Ir^4+^ ionization states within the coating achieve up to 25 mC/cm^2^ charge injection capacity at the interface of the coating (Merrill et al., 2005).

**Figure 1:**
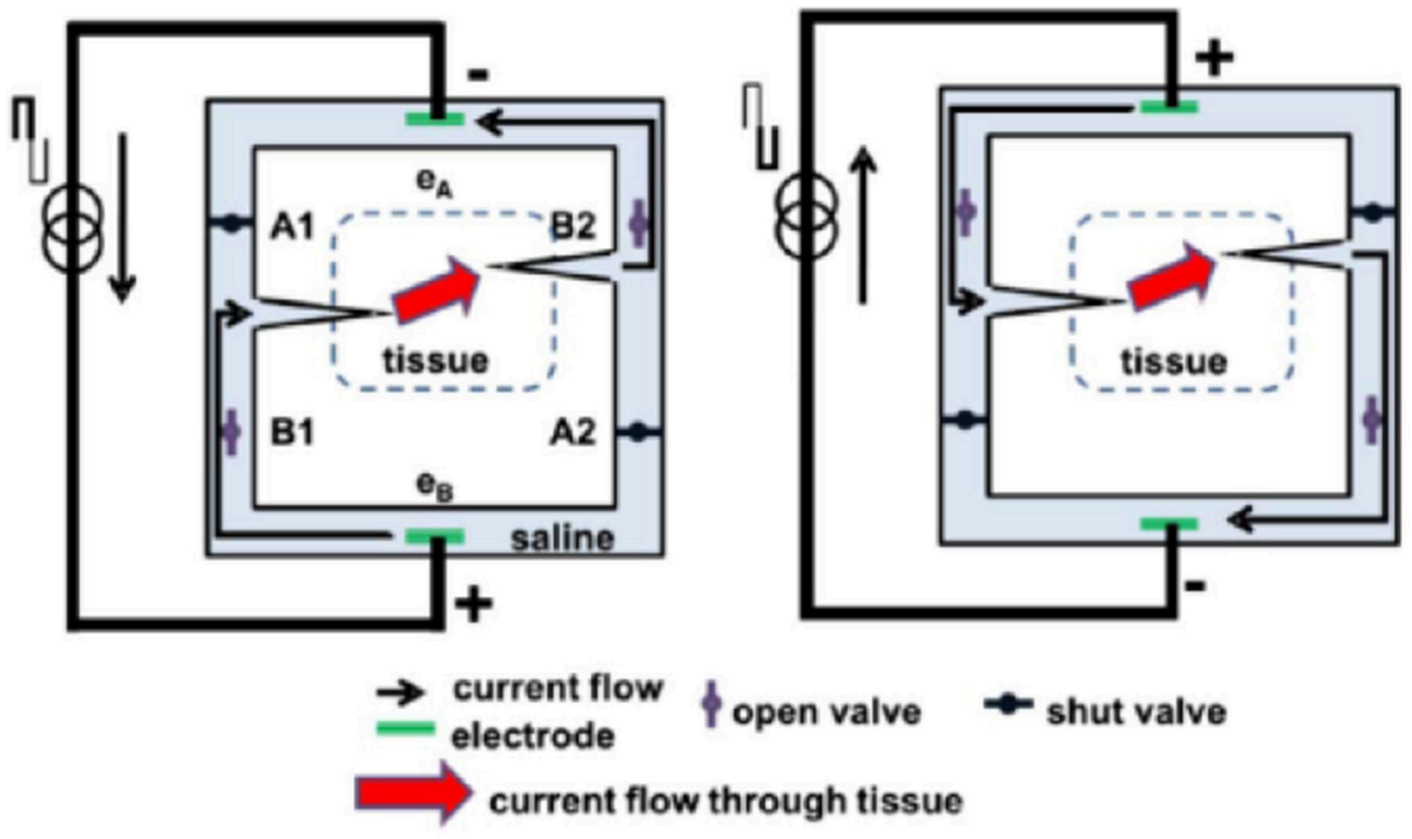
Principle of SDCS operation. The two diagrams indicate two states of the same device. The device converts electronic current delivered between electrodes e_A_ and e_B_ to ionic current delivered to the tissue. When the pulse switches current direction, the valves switch states and the ionic current is driven through the alternate paths of the bridge. In both cases the ionic current is driven from left to right. Figures adapted with permission from Fridman and Della Santina (2013a).

In contrast, another approach has been to develop a method by which ionic current can be delivered safely indefinitely (Fridman and Della Santina, 2013a,b; Ou and Fridman, 2017). The principle behind Safe Direct Current Stimulation (SDCS) is to rectify short, biphasic electronic pulses delivered to metal electrodes in the device into direct ionic current at the output of the device. This way the metal electrodes never undergo Faradaic reactions and there are no electrochemical byproducts generated within or external to the device. Conceptually, the SDCS delivers alternating current pulses to electrodes suspended at the opposite ends of a torus filled with ionic solution (termed “saline” in Figure 1). With each change in stimulation polarity the valves on either side of each electrode change from open-to-closed and closed-to-open, effectively modulating the path for ionic flow through the valves between low impedance and high impedance. Two extensions connected to the sides of the torus are directed into the body to complete the ionic current circuit. Figure 1 demonstrates this concept, comparing two states of the apparatus. In both panels of the figure, ionic current flows from left to right through the stimulated tissue. In this way, a continuous AC square wave controlling the apparatus will deliver DC ionic current (iDC) through the tissue from left to right. This system creates a closed-circuit path for the ions to flow, so that the anions that flow into the electrode tube on the right are replaced by the anions that flow out of the electrode tube on the left (Fridman and Della Santina, 2013a).

This concept originated from an attempt to develop a way to maintain endocochlear potential for hearing problems for people suffering from presbycusis, a common age related hearing disorder (Corbett and Clopton, 2004). This disorder disrupts the important ionic balance between endolymph and perilymph in the cochlea. The idea behind the system proposed by Spelman et al. was to create a mechanism by which electronic current delivered in the form of pulses could be rectified into ionic current flow. While this original application therapy was not developed further due to the advances in other treatments of age-related hearing loss, such as improved hearing aids and cochlear implants, this work did serve as an inspiration for developing the SDCS.

**Figure 2:**
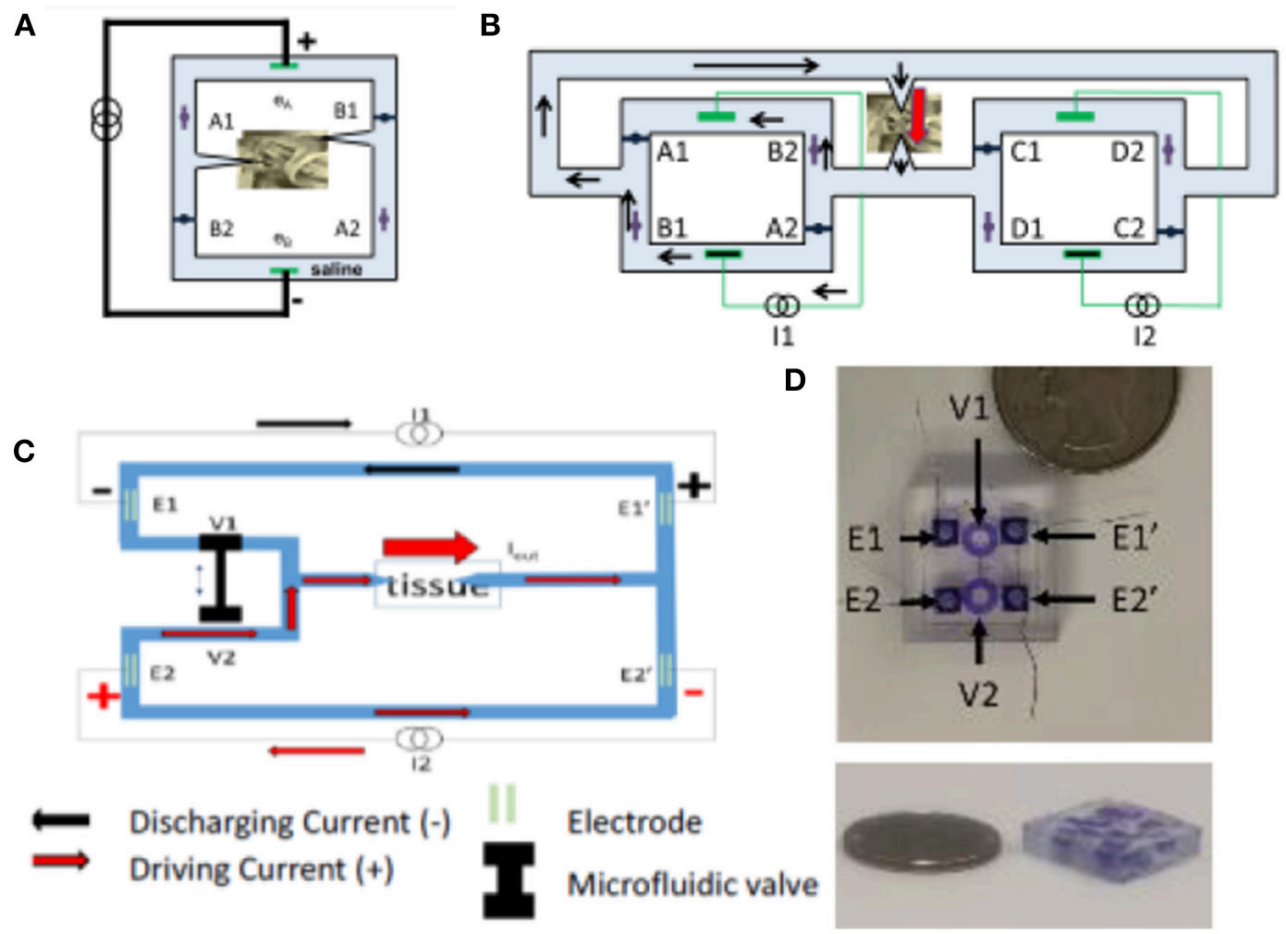
**(A)** SDCS 1. **(B)** SDCS2 was designed to remove interruptions in current flow observed in SDCS1 construction. **(C)** SDCS3 is designed to remove the current flow interruptions, reduce power consumption and improve reliability. **(D)** SDCS3 microfluidic implementation. Figures adapted with permission from Fridman and Della Santina (2013a) and Fridman (2017).

The SDCS has gone through several conceptual iterations and technological improvements. The original design shown in Figures 1, 2A, suffered from current flow interruptions due to valve transition timing. During state transitions, the valves would be closed or open simultaneously for a short duration at the same time, causing a short or an open circuit and resulting in interruptions in current flow at the output (Fridman and Della Santina, 2013a). The first solution to the problem of current flow interruption used two SDCS systems that worked in tandem shown in Figure 2B (Fridman and Della Santina, 2013b; Ou and Fridman, 2017). The system on the left would deliver the current to the tissue, while the system on the right would switch its valve states; the control of the current flow would switch electronically from the system on the left to the one on the right and the right system would change valve states, and then the process would repeat. Even though this solution solved the problem of current flow interruptions, the system suffered from high power requirements due to the need to operate eight independent valves.

The next design iteration addressed the problem of current flow interruption, and power consumption by reducing the number of valves to just two and requiring only one actuator to control these valves (Fridman, 2017). The basic construction is diagrammed in Figure 2C. Conceptually, this construction drives the current through the tissue using one current source, while the second discharges. During the valve switch, both valves are open for a short time, while the current sources ensure the proper amount of DC current flow through the tissue. The microfluidic prototype of this SDCS system is shown in Figure 2D. The valves are designed to be controlled on the PDMS chip using a shape memory alloy Nitinol muscle wire. The valves have been shown to operate for over 1 million cycles (Cheng et al., 2017, 2018; Fridman, 2017).

An alternative direction to mitigate electrical/biological interface concerns has been to develop an organic electronic ion pump (OEIP) that can deliver charged molecules directly from a reservoir to neural tissue via electrophoresis (Moulton et al., 2012; Arbring Sjöström et al., 2018). OEIP delivery of neurotransmitters has been shown to modulate neural activity both *in-vitro-* and *in-vivo-* (Isaksson et al., 2007; Simon et al., 2009, 2010, 2015), which could allow for OIEP-mediated neuromodulation to produce a more naturalistic control of neural activity. While OIEPs typically use DC current to drive electrophoresis, OIEPs are typically very low voltage and do not influence the membrane voltage of target neurons via a direct electric field effect, but rather by either the electrophoretic modulation of neurotransmitter or extracellular ionic concentrations (Simon et al., 2010; Larsson et al., 2013; Tarabella et al., 2013; Arbring Sjöström et al., 2018). Given the radically different mechanism of neural interaction that OIEPs employ compared to traditional or even ionic DC electrical stimulation, a full discussion of their mechanisms and potential applications is beyond the scope of this review. However, the reviews cited here discuss the development and function of OIEPs comprehensively (Svennersten et al., 2011; Moulton et al., 2012; Larsson et al., 2013; Arbring Sjöström et al., 2018).

## Electrical Stimulation—Neuron Interaction

With improvements to DC stimulation technology that reduce or even eliminate the safety concerns associated with toxic byproducts at the metal electrode-tissue interface, it becomes important to understand how the resulting focal iDC interacts with neurons. Unless explicitly mentioned, from here-on in the review it should be assumed that we are discussing only the effects of direct ionic current flow through the body and not the effects of undesirable electrochemical reactions at the tissue interface.

### Current Flow Through Tissue

The electric field associated with current delivered to an electrode in contact with the body depends on the impedance of the tissue through which this current travels. Even though the impedances differ greatly between different types of body tissues (Geddes and Baker, 1967; Pethig, 1987), each individual type of tissue impedance can be loosely modeled as a parallel resistor/capacitor pair, with the capacitance of the tissue modeling the impedance associated with the cell membranes, and the resistive components modeling the interstitial spaces (Gudivaka et al., 1999; Kyle et al., 2004). More accurately, finite element models (FEM) often include realistic morphologies of tissue and electric properties that include both conductivity and permittivity (Grant and Lowery, 2010; Joucla and Yvert, 2012; Wongsarnpigoon and Grill, 2012; Joucla et al., 2014). Capacitive and even dispersive impedances (in which permittivity varies as a function of frequency) are important when current propagation through tissue is pulsed at sub-millisecond duration (Grant and Lowery, 2010). These considerations are simplified for electrical stimuli at low DC-like frequencies, and can be reduced to models of purely resistive current propagation and static electric fields (Plonsey and Heppner, 1967). Just as for pulsatile stimuli, electric field orientations can be modified by introducing multiple electrodes, whose resulting electric fields would add in a standard linear superposition.

### Modeling the Effect of Direct Current on Neurons

Whereas, determining the electrical current propagation through the tissue is simpler for DC, the effect of DC electric field on neurons is more complicated when compared to the action potential (AP) evoked by a short biphasic pulse presentation.

The theory governing the effect of an extracellular electrode on neural membrane has been well-described using a compartmental cable equation that relates membrane-voltage to extracellular voltage and time-dependent membrane currents (McNeal, 1976; Rattay, 1986, 1999; Joucla et al., 2014). These equations were originally modeled on Hodgkin-Huxley or Frankenhaeuser-Huxley descriptions (Hodgkin and Huxley, 1952; Frankenhaueuser and Huxley, 1964), but the same compartmental model can be modified to rely on voltage-gated channel dynamics, based on the wealth of latest cellular electrophysiology literature—there are many types of voltage gated sodium and potassium channels, all with different temporal dynamics and expression densities, differentially expressed in the membranes of specific neural types (Vacher et al., 2008; Eijkelkamp et al., 2012; Toloza et al., 2018).

**Figure 3:**
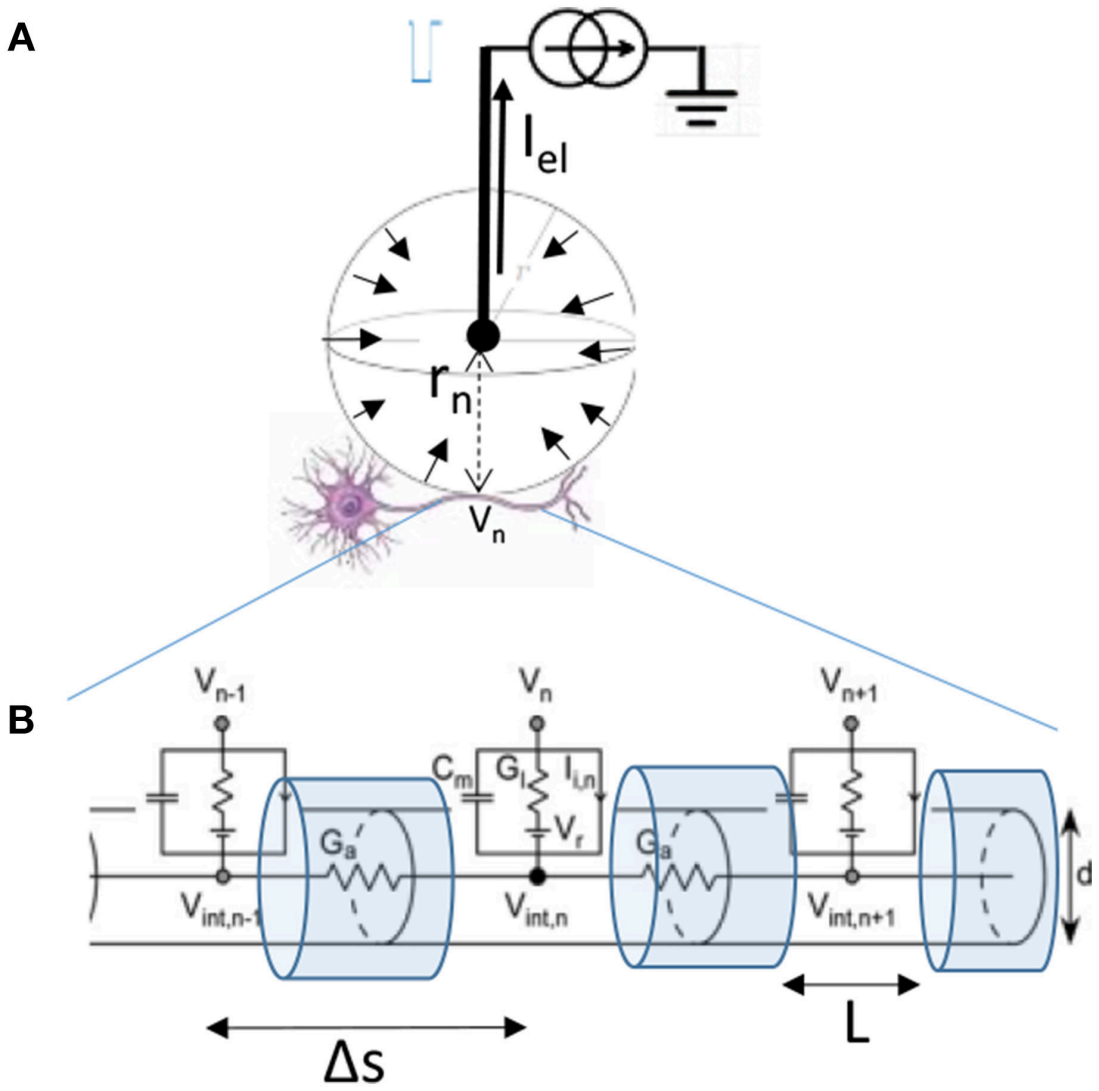
**(A)** Diagram of the single monopolar electrode distance r_n_ from the center of the targeted neuron. Because current travels to the electrode from all directions, we can assume that I_el_ is distributed over a sphere indicated by short arrows in an isotropic medium. **(B)** Myelinated axon of diameter d of the targeted neuron is modeled by compartments length Δs between nodes of Ranvier, with L representing the length of exposed membrane. Figure adapted with permission from Joucla and Yvert (2012).

For a monopolar electrode in an isotropic medium such as one diagrammed in Figure 3A, that delivers current *I*_*el*_ through tissue with resistivity ρ, the extracellular voltage *V*_*n*_ at distance *r*_*n*_ from the electrode is:

(1) $$\begin{array}{l} V_{n}=\frac{\rho I_{el}}{4\pi r_{n}}, \end{array}$$

The relationship that then relates the currents crossing the membrane of the targeted axon depicted in Figure 3B is the cable equation:

(2) $$\begin{array}{r} C_{m}\frac{d\left(V_{int,n}-V_{n}\right)}{dt}+G_{l}\left(V_{int,n}-V_{n}-V_{r}\right)+I_{i,n}\\ +G_{a}\left(2V_{int,n}-V_{int,n-1}-V_{int,n+1}\right)=0 \end{array}$$

Where the membrane conductances, currents, and capacitances depend on the diameter of the fiber d, the length of the segment Δs, and the exposed length of the membrane *L*. *C*_*m*_ = *c*_*m*_π*dL*, *G*_*l*_ = *g*_*l*_π*dL*, $G_{a}=\pi d^{2}/\left(4\rho_{i}\Delta s\right)$, *I*_*i,n*_ = *i*_*i,n*_π*dL*. *C*_*m*_ is the compartment membrane capacitance, *G*_*l*_ the membrane leakage conductance, *G*_*a*_ the conductance along the axon, and *I*_*i,n*_ the total transmembrane inward current (such as due to voltage gated channels). Membrane voltage (V_m_) can be expressed as the difference between the intracellular and the extracellular voltage *V*_*m,n*_ = *V*_*int,n*_ − *V*_*n*_. For further simplification, we can introduce the time and space constants: $\tau =\frac{C_{m}}{G_{l}}$, $\lambda =\Delta s\sqrt{\frac{G_{a}}{G_{l}}}$ (Joucla and Yvert, 2012). The equation can now be written as:

(3) $$\begin{array}{lll} \tau \frac{dV_{m,n}}{dt} & - & \lambda^{2}\frac{V_{m,n-1}-2V_{m,n}+V_{m,n+1}}{{\Delta s}^{2}}\\ \;+ & V_{m,n}+\frac{i_{i,n}}{g_{l}}=\lambda^{2}\frac{V_{n-1}-2V_{n}+V_{n+1}}{{\Delta s}^{2}} \end{array}$$

**Figure 4:**
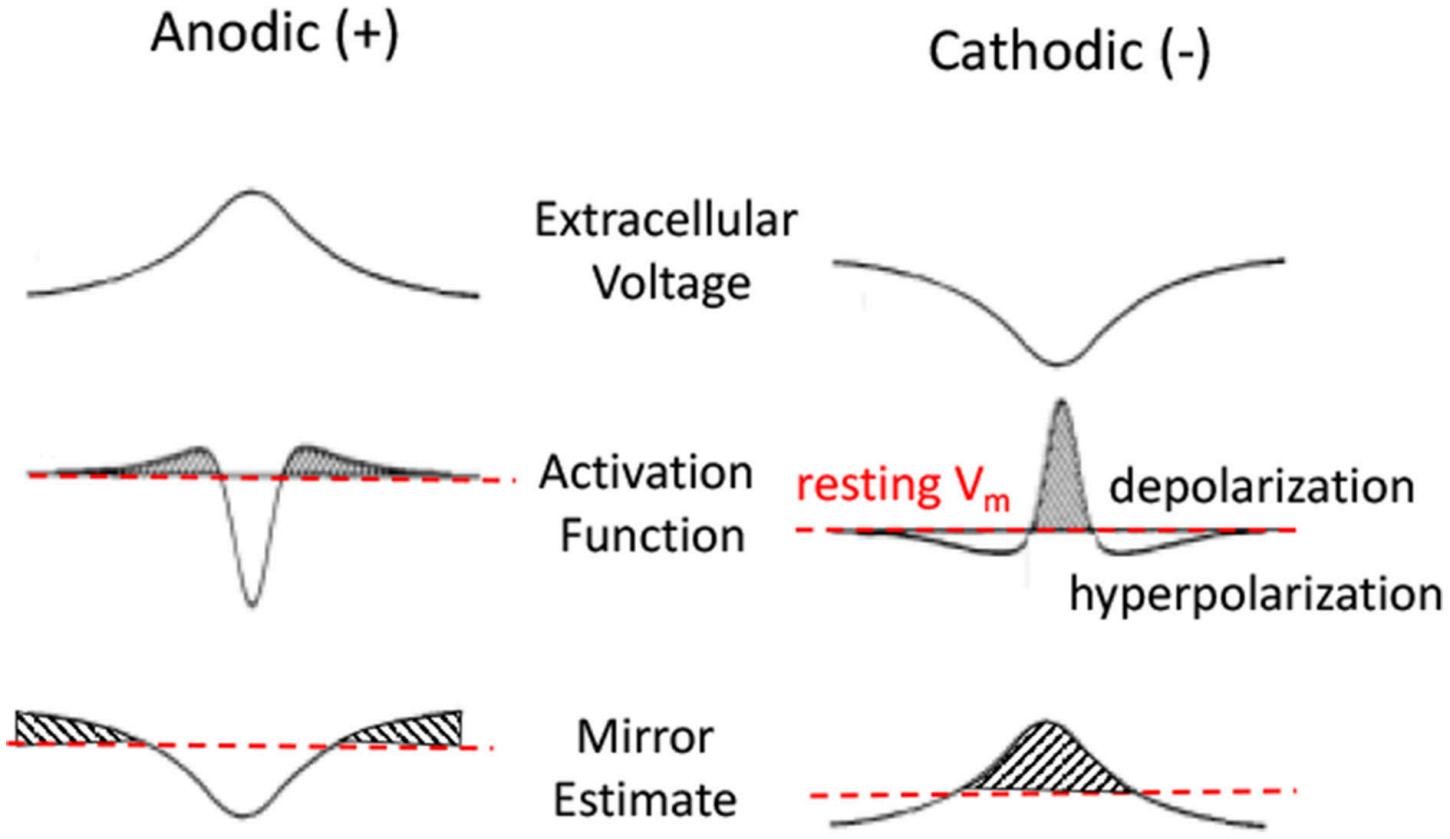
Anodic and cathodic stimulation model approximations. Top row is the extracellular voltage distribution along the fiber. Second and third rows are Activating Function (AF) and Mirror Estimate (ME) approximations of the resulting membrane potential changes. Anodic stimulation delivers a hyperpolarizing center, with short depolarized (shaded) side lobes. Cathodic stimulation delivers a strong depolarization near the electrode and hyperpolarization at the side lobes. The Activation Function is more accurate for short pulses. The Mirror Estimate is more accurate for long duration stimuli.

While the actual solution to this equation for a given stimulus is typically obtained by computational means it is possible to intuitively understand of the changes to the membrane potential *V*_*m*_ in response to a pulse presentation. Rattay introduced the concept of an Activating Function (AF) based on the cable equation (Rattay, 1986). For a rapid pulse presentation one can assume a dominant capacitive current across the membrane, negligible intracellular currents, and constant current through membrane channels. Under these conditions, he demonstrated that $V_{m}\;\alpha \;\frac{\partial^{2}V}{\partial s^{2}}\;$. That is, the membrane voltage is proportional to the second spatial derivative of the extracellular potential (Figure 4, second row). This approximation has been the primary tool used to predict the behavior of neurons in response to extracellular stimulation pulses (McIntyre et al., 2004). The drawback of this approximation is that it provides an intuitive description for the responses to very rapid sub-millisecond pulses, but not for the responses to long-duration stimuli such as those encountered during DC.

An alternative approximation, the Mirror Estimate (ME), was more recently proposed by Joucla and Yvert (2009). This approximation assumes a long-duration stimulus at which the capacitive currents become negligible and the membrane currents have reached a steady state. Using this approximation, it can be shown that the membrane voltage *V*_*m*_*(s)* at any point *s* along the fiber is proportional to the negative of the extracellular potential *V* offset from its average along the fiber: $V_{m}\left(s\right)\approx \bar{V}-V\left(s\right)$. The authors demonstrated this approximation to be more accurate over longer duration stimuli (Joucla and Yvert, 2012). This approximation is more relevant for estimating the effects of DC on the neural membranes.

Figure 4 shows an illustration of the predictions made by AF and ME approximations of the neural membrane response to a pulse presentation. The top row shows the extracellular voltage distribution along the horizontal axon for an electrode positioned in the center above the horizontal axon. The middle row shows the AF approximation (i.e., proportional to the second spatial derivative of the extracellular potential) of the membrane potential in response to a short pulse. The bottom row shows the ME approximation that is better at estimating the response for a long duration stimulus presentation. While AF and ME approximations of membrane voltage appear similar in shape, the ME has a much wider center, with considerably longer side lobes.

The illustrations for AF provide important intuitive predictions of the neural responses to a short pulse presentation. For example, from the second row, one can predict that a neuron will have a higher threshold for evoking an action potential to an anodic pulse presentation rather than to a cathodic pulse. This prediction is based on the fact that the anodic pulse depolarizes at the side lobes rather than in the center and since the side lobes are much smaller than the center response, one would need a larger amplitude stimulus to evoke an action potential. It is also apparent that the AP will be generated at the side lobes for an anodic stimulus, but it would be generated in the center for a cathodic stimulus. One can also predict that if the cathodic stimulus is large enough, the side lobes of the cathodic response (second row left) would prevent the AP from propagating from the center (Rattay, 1987).

### Predicting Neural Responses to Direct Current

Using the equations derived in the previous section, it is possible to make similar intuitive predictions about the types of neural responses generated in response to the long duration (DC-like) current as shown on bottom row of Figure 4. Here we present a set of predictions based on our examination of these ME approximations and in the subsequent section we explore the corresponding evidence from the empirical studies for DC effects on neurons. We describe the effects in terms of current amplitude, but these descriptions are equivalent if we describe the effects in terms of distance from the electrode as per Equation 1.

(a) When presented with a gradually increasing anodic stimulus, we would expect the membrane potential in the center of the axon to hyperpolarize enough that it would block or reduce the probability of AP propagation from its far distal end. (b) As the anodic stimulus amplitude increases further, we would expect to start depolarizing the side lobes enough to bring those parts of the membrane close to AP generation threshold and start to evoke its own action potentials.
(a) When presented with a gradually increasing cathodic stimulus, we would expect the membrane in the center to gradually become more depolarized, moving *V*_*m*_ closer to AP generation threshold, which will start evoking more action potentials from the center. (b) As the stimulus amplitude is further increased, we would expect that the side lobes will become sufficiently hyperpolarized to block or reduce the probability of AP propagation.
(a) We would expect the action potential propagation along the neuron to slow down during cathodic DC and (b) to speed up during anodic DC. The AP propagates from one segment (node of Ranvier) along the axon to the next by causing membrane voltage depolarization in the latter segment as a consequence of AP occurring at the previous neighboring segment. During the cathodic stimulus, the chronic hyperpolarization along the side lobes would require a longer time to force the membrane potential at each segment from their now more hyperpolarized state to AP generation threshold potential. The opposite would be expected from an anodic stimulus presentation.
In the low-cathodic or high-anodic DC delivery we would expect a more “natural” increase in neural firing rate. Because DC depolarization does not occur as a discrete event like a pulse, but instead creates a gradual persistent change in membrane potential, one would expect the resulting increase in firing rate to maintain the stochastic properties of the neuron's normal firing pattern. That is, for example if the neuron's action potentials are evoked distally as the result of excitatory postsynaptic potentials (EPSPs), we would expect increased AP generation because the new “resting” membrane potential is closer to threshold due to the extracellular DC delivery.
Steady state membrane potential should be significantly affected by the behavior of the membrane's ion channels during DC presentation. From this it follows that the specific types and densities of ion membrane channels are important to the membrane voltage change in response to the extracellular DC field. These considerations are typically ignored when the AF is used to predict the AP generation in response to a pulse presentation. This is because the sub-millisecond pulses are shorter than the changes needed to affect membrane channel conductances which occur on the order of milliseconds and longer.

### Experimental Observations of DC Neural Interaction

**Figure 5:**
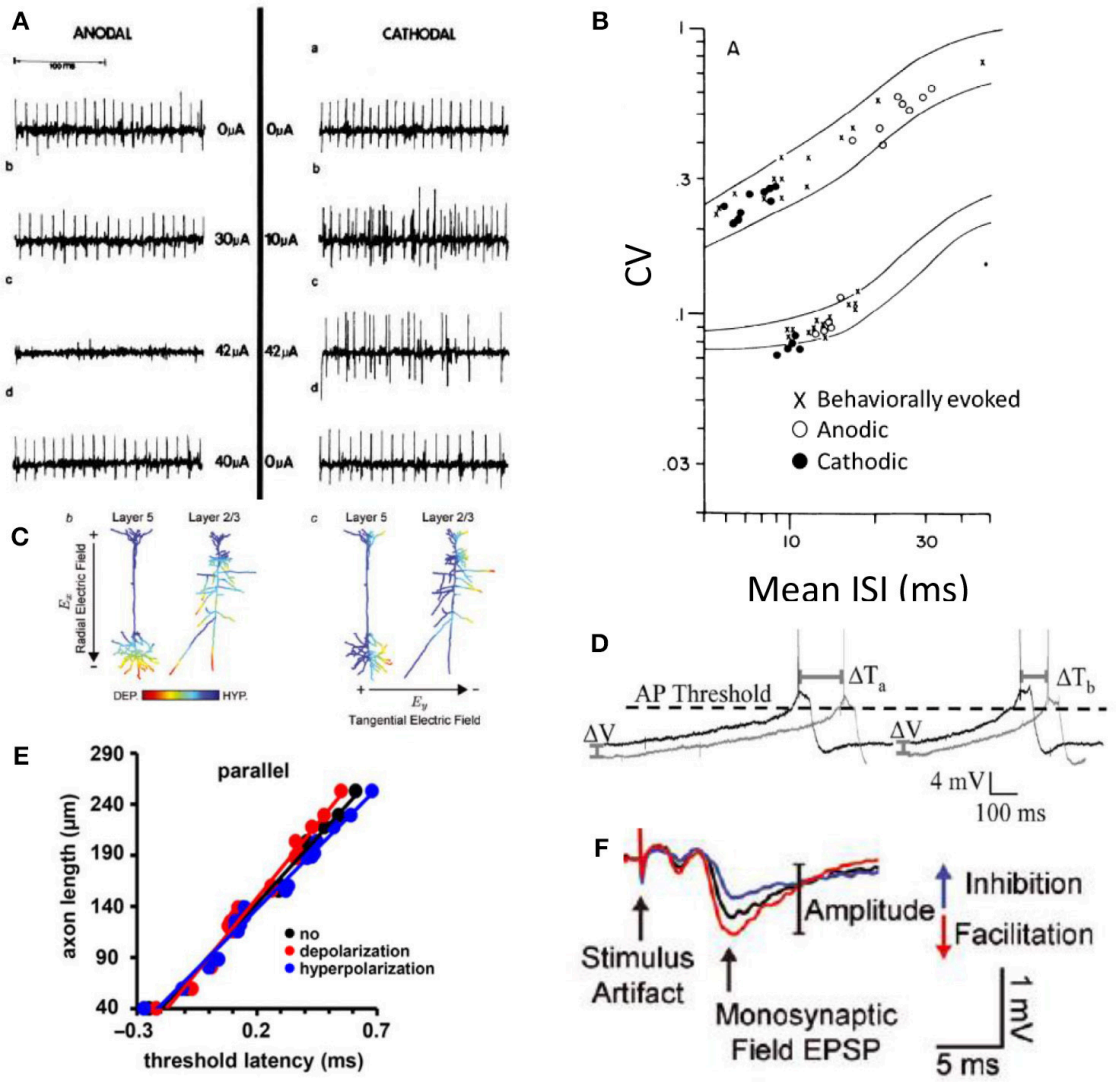
Experimental evidence for the predictions of DC effect on neurons adopted here with permission from the original publications. **(A)** Small cathodic (–) DC excites and small anodic (+) DC suppresses (Hopp et al., 1980). **(B)** Small cathodic DC excites and small anodic DC inhibits and the stochastic properties of each neuron are maintained as shown in the coefficient of variance (CV) measurement during DC for both cathodic and anodic stimuli (Goldberg et al., 1984). **(C)** Cortical neural responses to the direction of the DC field is consistent with a monopolar stimulation effect. Cathodic proximity excites (DEP) and anodic suppresses (HYP) (Rahman et al., 2013). **(D)** The timing of AP generation is affected by the DC field. Dark lines represent voltage changes during the application of the extracellular DC field (Radman et al., 2007). **(E)** Axonal latency of AP propagation plotted when the axon is parallel to the applied DC field. Red is cathodic, blue is an anodic stimulus. Slope of each fitted line corresponds to the conduction velocity in each condition (Chakraborty et al., 2018). **(F)** DC has a clear effect on the potentiation and depression of synapses as assayed here with an excitatory postsynaptic potential (EPSP) after exposure to the corresponding polarity DC fields (Rahman et al., 2013).

Because DC-based stimulation has not been on the forefront of neuromodulation research, relatively few electrophysiology experiments that assay single fiber responses to DC stimuli have been conducted. There is confirmation that low-anodic DC causes neural block and that low cathodic DC causes excitation. Figure 5A shows the anodal block and cathodal excitation at low amplitudes (10's of μA) in support of predictions 1a and 2a (Hopp et al., 1980). In this experiment, DC stimulation was delivered *in-vivo* to the middle of a dog vagal nerve axon while AP propagation was assayed via periodic distal depolarization. Further support for predictions 1a, 2a, and prediction 4 was obtained in a chinchilla vestibular nerve experimental results shown in Figure 5B (Goldberg et al., 1984). Vestibular afferents normally maintain spontaneous activity at ~60–100 spikes/s. In this experiment, vestibular afferents were being recorded during application of chronic DC stimulus. In this plot, the responses of two types of afferents are shown. The regular fiber maintains nearly constant inter-spike intervals (lower stripe), while the irregular fiber (upper stripe) does not. As expected from prediction 2a, cathodic stimulus (black dots) increased the firing rate [shown as decrease in the inter-spike interval (ISI)] of the afferent neuron. Also, in agreement with prediction 1a, Anodic stimulus (open circles) decreases firing rate. Furthermore, illustrating prediction 4, the coefficient of variation (CV) that describes the regularity of inter-spike intervals is maintained during DC stimulation when compared to those from behaviorally (not electrically) modulated spike rates (Goldberg et al., 1984).

An increase and decrease of neural activity using correspondingly small cathodic and anodic currents (predictions of 1a and 2a) is further supported by a functional demonstration of DC neuromodulation, also conducted in the vestibular system (Aplin et al., 2018). The results of this work also suggest that DC modulation does not cause entrainment of activity, consistent with prediction 4. Previous experiments implicated phase-locked synchronized multi-neuronal responses to the vestibular nerve pulse-train presentations in the subsequent long-term depression of the synapses in central nervous system (Mitchell et al., 2016, 2017). In contrast to pulsatile train modulation of the nerve, DC modulation did not appear to cause the same depression of sensitivity, although these results are not directly comparable (Aplin et al., 2018).

The effect of anodic and cathodic block on efferent peripheral fibers has been investigated more thoroughly (Bhadra and Kilgore, 2004; Vrabec et al., 2016, 2017), and have concluded that monopolar block can be achieved with both DC polarities in support of predictions 1a and 2b, and 5. A surprising finding in these investigations is that a cathodic block could occur at the center part of the axon closest to the monopolar source, which should be depolarized as seen in Figure 4 bottom row right. The authors' explanation for how this block could occur is that during cathodic DC application, the voltage gated sodium channels are held in their inactivated states, preventing AP propagation (Bhadra and Kilgore, 2004). This argument is further supported by the investigation of the peripheral afferent cathodic block (Yang et al., 2018).

In agreement with predictions 3a and 3b, the timing and shape of AP generation is affected by DC fields (Shu et al., 2006; Radman et al., 2007). The timing of action potential propagation is affected with DC fields as shown in Figures 5D,E. Figure 5D shows the action potential being generated more rapidly due to increased membrane voltage slope affected by the DC field. Figure 5E shows the latency associated with AP propagation when anodic or cathodic DC stimulation is applied. The slopes of the lines indicate conduction velocity. Anodic DC increases the speed of AP propagation and cathodic DC decreases it (Chakraborty et al., 2018).

Transcranial direct current stimulation (tDCS) has been shown to have significant clinical implications for psychiatric disorders, epilepsy, stroke, Parkinson's disease, and pain suppression (Jackson et al., 2016). While the mechanisms are not well-understood, it is clear that cortical neurons subjected to typical tDCS electric fields experience only very subtle sub-millivolt changes to their membrane potentials—much lower than the 10 s of millivolts necessary to evoke action potentials. The effects of DC are therefore much more subtle and likely involve synaptic modifications through controlling spike propagation timing and consequently spike-timing dependent plasticity (Radman et al., 2007). Isolated hippocampal slice experiments that exposed neurons to weak directional DC fields identified several important effects of DC on neural signaling. One key finding in these experiments is that orientation of the electric field matters. This field orientation is consistent with the monopolar stimulation concept that we described earlier. The part of the axon closest to the anodic (positive) electrode will be more hyperpolarized than the parts that are farther away (Figure 5C). If the electric field is oriented parallel to the fiber, the fiber's response is affected. If it's perpendicular, there's no effect on the fiber's membrane potential (Bikson et al., 2004; Rahman et al., 2013; Jackson et al., 2016; Chakraborty et al., 2018).

When synaptic transmission is occurring, the synaptic connections are potentiated or depressed depending on the orientation of the electric field. The effect of DC fields on synapses is shown in Figure 5F with the size of the excitatory postsynaptic potential after cathodic and anodic DC fields are applied to the synapse. If the field is oriented such that the cathode is on the postsynaptic end of the synapse, it will be depressed. Conversely, if the anode is positioned toward the postsynaptic end of the synapse, it will be potentiated (Bikson et al., 2004; Kabakov et al., 2012; Rahman et al., 2013; Jackson et al., 2016).

In support of prediction 5, ionic channel conductances during DC presentation appear to critically influence the neural response (Yang et al., 2018). This was indicated with a neural block experiment in which a cathodic block exhibited different thresholds on different neuronal fiber types, which could not be explained entirely via differences in fiber diameter. Additional evidence for the strong effect of membrane channel dynamics in a DC field comes from an examination of network responses: the modeled effect of DC on neural network responses matches the observed effect only when hyperpolarization activated cation current dynamics (I_h_) are included in the model (Magee, 1998; Toloza et al., 2018).

## Potential Applications for DC Modulation

As discussed in the previous section and summarized in Table 1, DC can increase or decrease firing rate, block AP propagation, potentiate and depress synaptic connections, and increase or decrease neural conduction velocity. The parameters that control how DC interacts with neurons are the position, polarity and the amplitude of the electric field. It is clear that DC modulation offers the ability to interact with the nervous system in many new ways.

**Table 1:** Summary of DC effects on neurons from both intuitive predictions suggested by the Mirror Estimate model and experimental evidence.

**Prediction of DC effect on neural signaling**	**Neurophysiological application importance**	**Experimental evidence**
1a. Low anodic (+): block of AP propagation	Pain propagation block, urinary bladder control, vestibular prosthesis	Hopp et al., 1980; Goldberg et al., 1984; Bhadra and Kilgore, 2004; Vrabec et al., 2017; Aplin et al., 2018
1b. High anodic (++): increased firing rate		
2a. Low cathodic (–): increased firing rate	Vestibular prosthesis, other conventional excitatory neuromodulation paradigms that currently use pulsatile stimuli	Hopp et al., 1980; Goldberg et al., 1984; Aplin et al., 2018
2b. High cathodic (– –): block of AP propagation	Pain propagation block, urinary bladder control	Bhadra and Kilgore, 2004; Vrabec et al., 2017; Yang et al., 2018
3a. Anodic (+): increase of AP conduction velocity	Synaptic connectivity, LTP, LTD, CNS modulation	Radman et al., 2007; Chakraborty et al., 2018
3b. Cathodic (–): decrease of AP conduction velocity	Synaptic connectivity, LTP, LTD, CNS modulation	Radman et al., 2007; Chakraborty et al., 2018
4. Stochastic firing properties are maintained under DC excitation and inhibition	Synaptic connectivity, LTP, LTD, CNS modulation	Goldberg et al., 1984
5. Types and dynamics of ionic conductances play an important role in membrane voltage changes during extraceIIular DC stimulation	Synaptic connectivity, LTP, LTD, information processing, fiber selectivity, CNS modulation	Shu et al., 2006; Chakraborty et al., 2018; Toloza et al., 2018; Yang et al., 2018

In the following discussion we focus on potential applications for DC neuromodulation in systems that can potentially stand to benefit from the unique aspects of DC modulation.

### DC for Inhibition/Block of Peripheral Pain

Electrical neuromodulation is a clinical intervention used to treat pain after unsuccessful pharmacological therapy. Although there are multiple approaches to the electrical modulation of pain, typically varying by the location of current delivery, they all rely on pulsatile electric stimuli to disrupt afferent pathways delivering nociceptive information to the brain (Gofeld, 2014; Geurts et al., 2017; Krames et al., 2018). The ultimate aim of neuromodulation of pain is to block undesirable nociceptive input; this is problematic for pulsatile stimulation as it cannot directly suppress neural firing and must indirectly suppress nociceptive circuits via lateral inhibition or disrupt them via the delivery of high-frequency modulation. Trials assessing the efficacy of pulsatile electrical neuromodulation of pain relief have mixed results and report side effects such as undesirable hypersensitivity or somatosensory sensation (Nnoaham and Kumbang, 2008; Geurts et al., 2013, 2017; Shamji et al., 2017). Direct current modulation may hold a potential solution for these issues. DC can suppress or completely block nociceptive fibers directly, in principle foregoing the need for indirect or alternative approaches for suppression of the target neurons (Bhadra and Kilgore, 2004; Vrabec et al., 2017). DC fields may also be able to better target the thin myelinated Aδ fibers and unmyelinated, small-diameter C fibers that are the primary transmitters of the nociceptive signal in the periphery (Yang et al., 2018).

There have been several approaches to the development of DC-mediated pain suppression. Charge balanced DC delivery via an implanted microfluidic device (Fridman and Della Santina, 2013a; Cheng et al., 2017; Ou and Fridman, 2017) could potentially deliver chronic inhibition or block to peripheral nerves (Yang et al., 2018). High capacitance electrodes for sustained DC block and a separated interface system could be used to more efficiently deliver temporary DC pain suppression (Ackermann et al., 2011; Vrabec et al., 2016, 2017). An alternative percutaneous approach has also been developed to both suppress pain and potentially encourage wound healing in traumatic injury (Molsberger and McCaig, 2018). These technologies remain in pre-clinical development but have promising results and are likely to be among the first DC-based neuromodulation therapies to attempt clinical translation.

### DC for Modulation of the Vestibular Periphery

Another system that could benefit from the ability of DC modulation to inhibit neural activity is the vestibular system. In a normally functioning system, the semicircular canals of the vestibular labyrinth encode the direction and magnitude of head rotational velocity by increasing or decreasing firing rate from a spontaneous baseline of approximately 100 spikes per second in primates (Miles and Braitman, 1980; Sadeghi et al., 2009). In patients with bilateral loss of vestibular function it becomes debilitatingly difficult to maintain stable vision, balance or locomotion (Curthoys and Halmagyi, 1995). Vestibular prostheses modeled on the cochlear implant are in development to address a therapeutic need in patients with vestibular pathologies (Della Santina et al., 2007; Valentin et al., 2013; Hageman et al., 2016; Lewis, 2016). To generate bidirectional encoding of motion, these implants artificially elevate the spontaneous rate of the vestibular afferents via adaptation to a constant pulse rate and then modulate around this increased baseline. This approach has two main limitations: the first is that an increased baseline rate reduces the dynamic range of encodable head rotations (Davidovics et al., 2011, 2012; Lewis, 2016) and the second is that entrained firing of the vestibular nerve (a natural consequence of pulsatile stimuli) is thought to result in long-term depression of post-synaptic neurons in the vestibular nuclei (Lewis, 2016; Mitchell et al., 2016). Direct current can address both of these limitations, as it can both excite and inhibit neural activity as well as deliver “naturalistic” changes in firing rate that retain stochastic variability (Goldberg et al., 1984). iDC modulation using ionic conduits implanted into the semicircular canals of the chinchilla can produce a greater dynamic range of reflexive eye rotations when compared to a pulsatile stimulus, without suppression at higher current baselines (Aplin et al., 2018). Further work will be necessary to test mechanism of action and to demonstrate chronic efficacy and safety of an ionic DC-based vestibular implant.

### DC Stimulation for Bladder Control

A common consequence of spinal cord injury is difficulty controlling micturition (urination) reflexes (Krause et al., 2004). Typically, patients suffer from an inability to fully void the bladder, which can lead to severe complications including kidney failure (Krause et al., 2004; Biering-Sørensen et al., 2008). Non-invasive DC modulation of the spinal cord has had some success restoring micturition function in humans (Flamm et al., 1977; Radziszewski, 2013; Stewart et al., 2016; Zhu et al., 2016). The mechanism of action is thought to be subthreshold modulation of spinal circuits rather than the result of DC-evoked spike trains (Kerns et al., 1996; Ahmed and Wieraszko, 2012; Ahmed, 2013, 2017). The current focus has been on non-invasive DC delivery due to the historical inaccessibility of invasive DC delivery. This field might thus stand to benefit from the development of safer DC delivery *in-vivo-*: in the human, non-invasive trans-spinal DC affects a relatively large and non-uniform area (Kuck et al., 2017), while invasive trans-spinal DC has the potential to deliver current to a much more confined target. DC delivery closer to the target tissue could improve effect thresholds and reduce current spread to non-target tissues. Additionally, non-invasive DC still must contend with toxic electrochemical reactions generated at the metal-electrolyte interface, and is typically only delivered therapeutically for relatively short periods (Radziszewski, 2013). An implantable system capable of delivering chronic DC could provide tonic active therapy for cases where short-duration therapy is not efficacious.

### Cortical Modulation Using Invasive DC

The potential for non-invasive (trans-cranial) DC to influence cortical firing via diffuse, sub-threshold electric fields has seen a great deal of recent interest and active research (Ruffini et al., 2013; Bikson et al., 2016; Jackson et al., 2016). Invasive DC has the advantage of being able to deliver higher and much more locally directed electric fields, but the use of invasive electrotherapy in the brain is harder to justify given the difficulties and significant safety concerns associated with invasive penetration into the CNS. As invasive DC technology matures, there may be opportunities to address neural targets within the brain, particularly where pathological conditions occur as a result of hyperexcitability, such as in epilepsy. Locally delivered DC modulation can down-modulate neural activity and has been shown *in-vitro* to suppress epileptiform activity in the rat hippocampus (Lian et al., 2003). Invasive electrotherapy is already used for suppression of epileptic activity when other treatments have failed (Stefan and Lopes da Silva, 2013; Mina et al., 2017) and non-invasive DC is an emerging therapy for epilepsy although the extent of efficacy is controversial (San-juan et al., 2015). However, invasive DC modulation will first have to prove significant potential benefit over preexisting non-invasive therapies for epilepsy to be considered as a viable alternative approach.

Another potential cortical target for invasive DC is the treatment of chronic tinnitus. Tinnitus is characterized by persistent auditory illusion and is widespread with limited available treatment options (Møller, 2007b). Etiology in tinnitus is extremely varied, but some forms of the condition are thought to arise due to corruption of auditory processing at the cortex (Møller, 2007a,b; De Ridder et al., 2014). Some of the primary effects of tonic, subthreshold DC fields are changes in excitability and conduction velocity, which can create plastic changes in cortical networks (Radman et al., 2007; Jackson et al., 2016). Non-invasive DC has been explored as a potential therapy for tinnitus, but existing evidence suggests it is not consistently efficacious at reducing symptoms for most patients (Song et al., 2012; Santos et al., 2018). One problem is that neural changes in tinnitus may be highly heterogeneous across local cortical regions (Møller, 2007a; De Ridder et al., 2014). We hypothesize that locally delivered DC has the potential to deliver much more constrained electric fields and thus might provide extra benefit over diffuse non-invasive fields, but studies on animal models of tinnitus will be necessary to test the validity of this hypothesis.

### Cochlear Implants

Cochlear implants (CI) are the great success story of the field of neuromodulation. Neuromodulation via CI is now a common treatment for severe hearing loss, with bilateral implantation becoming almost routine. Most people hear with the CI well-enough to talk on the phone in a quiet room. While there's no debate regarding the usefulness of these devices in everyday life, significant hearing deficiencies with the CI in presence of noise, tonal language speech perception (such as Mandarin Chinese), and poor music perception still remain as issues to be addressed (Wilson and Dorman, 2008; Prevoteau et al., 2018).

One potential explanation for the difficulty to convey musical melody is that the cochlear implants typically deliver pulses using some variant of a continuous interleaved stimulation (CIS) paradigm. This method of stimulation delivers pulses in a round-robin sequence to each electrode along the implanted array. With the traditional CIS, each electrode is pulsed at approximately 250–1,000 pps (An et al., 2007; Wilson and Dorman, 2008). Sound information is introduced by varying the amplitude of the delivered pulses proportional to the power of the sound localized by its frequency band. This stimulation method is preferred in cochlear implants because it clearly reduces hearing noise due to channel interaction by allowing targeted populations of neurons to respond without interference from the neighboring channels (Wilson et al., 1991).

In normal hearing, the afferents that carry the sound information to the brain can be divided by their spontaneous activity profile. There are the high spontaneous, and low spontaneous activity neurons. All of these neurons modulate their firing rate depending on the amplitude of the signal within the band. High spontaneous fibers have lower thresholds and sharp rise in firing rate with increased sound amplitude. The low spontaneous fibers have a higher threshold and a very slow-rise linear relationship between firing rate and sound amplitude (Kiang et al., 1976; Liberman, 1978).

From the perspective of neural firing, while the CIS coding scheme recruits different populations of neurons depending on the amplitude of the pulse, recruited neurons all respond in synchrony with high spike rates that correspond to the constant pulse rate delivered from the pulse generator. Thus, the louder the sound within the frequency band, the more neurons fire at high constant spike rate.

This method of stimulation bypasses the linear relationship between firing rate and sound amplitude and compresses the entire range of response to an almost a binary signal for each responding neuron. Using cathodic DC amplitude to encoding sound could reintroduce the more natural recruitment of firing rates in proportion to sound amplitude.

## Non-Metal-Electrode Based DC Safety Considerations

As DC-enabling technology progresses toward chronic implantation, it will become increasingly important to characterize how DC fields affect the physiological and electrochemical environment to which they deliver their charge. Emerging technologies provide a potential means to mitigate or eliminate the build-up of toxic electrochemical reactions associated with DC stimulation due to a lack charge recovery at the metal-tissue interface (Ackermann et al., 2011; Fridman and Della Santina, 2013a). However, even charge balanced current delivery is capable of damaging tissue when the total charge or charge-per-phase is large (Brummer and Turner, 1977; McCreery et al., 1990; Merrill et al., 2005; Cogan et al., 2016). Many of the safety limitations for a DC-based implantable device, such as electroporation thresholds, are likely to be a function of total charge delivered (Merrill et al., 2005; Nakauchi et al., 2007; Cogan et al., 2016) and thus similar to those of an IPG-based device. Given that there are already many reviews addressing safe charge densities in IPGs, this section will instead focus on potential safety considerations that have a unique aspect specific to tonic DC delivery.

### Temperature

Biological tissue has an impedance associated with it which varies depending on the current path through the tissue (Foster and Schwan, 1989; McAdams and Jossinet, 1995; Gabriel et al., 1996). When electricity travels through a resistance, some of the energy of the current is converted into heat via a process called joule heating, with power as a function of the resistance and current: P = I^2^R. Even relatively small changes in temperature can impact neural function (Hodgkin and Katz, 1949) with an increase of 4–5°C resulting in rapid neuronal death (Wang et al., 2014). Like any other device that passes current through the body, implantable DC devices must therefore consider the effect of joule heating on both acute and chronic device safety. It might seem intuitive to consider tDCs safety margins as a close analog to an implantable DC device, but joule heating effects are not comparable between the two methodologies: tDCs must address larger currents and dermal resistances but also benefits from a greater surface area and distance from the target tissue (Datta et al., 2009; Bikson et al., 2016; Gomez-Tames et al., 2016). Implantable devices using DC in the body differ from IPGs due to the lack of phase associated with DC. This has two primary effects: the first is that, for a given current amplitude, DC can potentially deliver a greater charge density, which directly impacts the amount of joule heating produced per unit time. The second effect is that tissue impedances are a function of both the resistance and capacitance of the tissue (typically visualized as a resistor and capacitor in parallel) and, because DC has no frequency, impedances are thus typically higher for DC delivery than for an equivalent high frequency AC waveform (Foster and Schwan, 1989; McAdams and Jossinet, 1995; Faes et al., 1999). In contrast, lower amplitude thresholds for implantable DC devices may offset the higher durations of delivery. For example, a recent comparison of behavioral responses for DC and AC stimulation waveforms of the peripheral vestibular system (Aplin et al., 2018) found that amplitudes for DC waveforms are typically less than for AC waveforms. Similar impedances and peak behavioral responses could be achieved using either 40 μA DC or 420 Hz, ~175 μA/phase, 400 μs/phase biphasic pulses. The total charge delivered per second for both waveforms is comparable: 40 and 29.4 μC/s for DC and AC waveforms, respectively. As with any electrically active device implanted in tissue, invasive devices that deliver DC will have to show that local thermal changes remain in a range (< ~2°C) where the effects on neuronal activity are comparatively mild (Wang et al., 2014).

### Electroporation

Electroporation is the process of pore formation in the lipid bilayer of cells membranes when subjected to an electric field (Bramlet, 1998). Irreversible electroporation occurs when this process leads to formation of permanent fenestrations in the membrane and subsequent loss of ion homeostasis and cell death (Rubinsky, 2010). Electroporation thresholds are a function of field density with thresholds in the range of 1 kV/cm and can be achieved by both pulsed and DC fields (Bramlet, 1998; Kim et al., 2007). Cell death due to irreversible electroporation can occur as an unintended side-effect of pulsatile electrical stimulation with a threshold relating to both the total charge density and the charge delivered per pulse phase (Butterwick et al., 2007). Electroporation thresholds are typically well above the range of functional IPG stimulation (Merrill et al., 2005; Butterwick et al., 2007; Nakauchi et al., 2007; Boinagrov et al., 2010). There is a non-linear relationship between pulse width and amplitude threshold for electroporation thresholds, with charge delivered over a longer unit time resulting in higher thresholds when compared to the same charge delivered over a short period (Butterwick et al., 2007; Nakauchi et al., 2007; Boinagrov et al., 2010). This may lead to comparatively higher electroporation thresholds for DC because, even though charge delivery per second might be higher, peak charge is much lower.

For example, an implantable DC neuromodulation for pain would be expected to be delivered at a maximum 10 mA (Vrabec et al., 2016, 2017; Yang et al., 2018). The worst-case assumption is that the current is driven in a straight line through a nerve, rather than spread through a homogeneous environment like the brain. Ten milliampere current driven through nerve tissue of 1 kΩcm specific resistance will produce only 10 V potential over a cm of tissue. This voltage is 100 times lower than the typical 1 kV/cm electroporation threshold. It is therefore unlikely that electroporation would result from DC neuromodulation.

### pH Changes

IPGs typically only generate transient pH changes (<1 unit) within a small (~1 μm) radius at the tissue-electrode interface (Ballestrasse et al., 1985; Huang et al., 2001; Chu et al., 2004) and, unless the device is malfunctioning, are not considered to be a significant safety concern (Merrill et al., 2005). While DC electrolysis will, by definition, generate large pH changes between the cathode and anode, proposed DC implantable technologies mitigate this by reversing electrolytic reactions via charge balancing the metal electrodes (Fridman and Della Santina, 2013a) or by chemically and physically buffering the electrode/tissue interface (Ackermann et al., 2011). Ionic DC delivery could still influence pH at the microfluidic/tissue interface as sustained ionic current acts as an ion pump that over time might change the H^+^/OH^−^ concentrations at the tissue/device interface, and a locally sustained electric field can create a pH gradient even in the absence of electrolysis (Macounová et al., 2000; Berkelman, 2005). Given that these effects have historically been masked by pH changes from DC electrolysis there has not yet been an exploration of their significance *in-vivo*. We hypothesize that the theoretical pH gradients generated by ionic DC would be mild and easily compensated for by e.g., natural diffusion and acid-base homeostasis (Hamm et al., 2015) particularly given that neurons are resistant to small (<1 unit) changes in pH (Goldman et al., 1989). Ultimately, the magnitude of potential pH changes in ionic DC fields will have to be confirmed in preclinical safety experiments.

### Electrophoresis

Electrophoresis is the movement of charged particles in a fluid as the result of an electric field. In fluids, current is propagated via the physical movement of charged species (ions) through the conductive media. In biological tissue, these species are predominantly Na^+^, K^+^ and Cl^+^, but also any other charged molecule including large protein complexes—this has long been exploited by biologists to separate proteins on the lab bench (Abramson et al., 1942). The movement of ions is dependent on the size and mass of the ion, the density of the media, the charge of the ion and the strength of the electric field, with typical flow speeds of small ions in the range of ~100 μm/s for a 100 V/cm field in an unobstructed media. Electrophoresis is not normally a consideration for neural stimulators: during pulsatile stimulation, charge balanced waveforms ensure virtually no net movement of charged molecules. In contrast, DC waveforms are not charged balanced and will cause electrophoretic transport of ions over time. It is known that the body utilizes electrochemical gradients to transport charged proteins (Jaffe, 1977) and it has been proposed that non-invasive DC may have electrophoretic effects on neural tissue (Rae et al., 2013), so there is at least some evidence to suggest that electrophoretic mechanisms should be considered when designing chronically stimulating DC devices. For example, if the ionic composition at the cathode/anode are not identical to the physiologic medium it might be possible to locally deplete a charged protein or ion by “pumping” ions from the microfluidic device to the tissue and displacing preexisting ionic concentrations. This can be mitigated by ensuring that the microfluidic system supplying ionic DC to the tissue has an ionic composition similar to the extracellular medium, and by placing the anode/cathode in locations with similar interstitial ionic concentrations.

## Tissue Effects of Sustained Electric Fields

Neural tissue is the most electrically active tissue in the body and the typically the primary target for therapies involving electrical stimulation, including those discussed in this review. Endogenous electric fields also occur naturally within other tissues in the body, and these tissues can have electric-field mediated responses, particularly for directing cell migration during development or wound healing (Bramlet, 1998; McCaig et al., 2005). Sustained electric fields may also influence the distribution of charged proteins, membrane potentials and pH gradients in otherwise electrically inactive tissues (Abramson et al., 1942; Jaffe, 1977; Funk, 2015). Locally delivered DC fields via an invasive device may avoid some of these considerations due to a finer control of field distribution, but as the intention may be to deliver chronic DC current over the lifetime of the device, focal effects may be stronger than what might be expected in e.g., tDCs where treatment times are typically very short (5–30 min vs. potentially years or decades for an implanted device). This section briefly outlines several effects of DC electric fields on non-neural tissue and discusses their potential relevance to chronic DC neural stimulation.

### Neural Migration and Axonal Growth

Endogenous electric fields are an important mechanism in the developing nervous system to facilitate differentiation and stratification of nervous tissue, and in the mature animal in response to nervous tissue damage (Mccaig and Rajnicek, 1991; McCaig et al., 2005). Artificially generated electric fields have been shown both *in-vivo* and *in-vitro* to direct stem cell migration (Yao and Li, 2016; Feng et al., 2017), axonal growth (Yamashita, 2015), and may also influence stem cell differentiation (Zhao et al., 2015). Typically, in a DC field, axonal growth or cell migration is attracted toward the cathode and repelled away from the anode, although this can vary depending on the neural type and substrate (Mccaig and Rajnicek, 1991). Axonal and cell growth can be influenced *in-vitro* with sustained electric fields on the order of ~10–100 mV/mm (Mccaig and Rajnicek, 1991).

The effect of polarity-dependent axonal growth is an important consideration for the functional modulation of nervous tissue with DC. If the modulated field is excitatory it might encourage axonal growth near the implantation site (Fehlings et al., 1989). An inhibitory field may have an opposite effect—which could be beneficial if the aim is to reduce overall connectivity in the area but could also potentially cause axonal retraction and thus increased stimulation thresholds. It is difficult to predict how these effects may interact with ongoing degeneration of a neural substrate or the regions of inflammation and glial scar formation that tend to surround implanted neural stimulators *in-vivo* and reduce their efficacy over time (Szarowski et al., 2003; Cicchetti and Barker, 2014; Groothuis et al., 2014). Careful consideration of these effects will be an important component of any long-term histological assessment of implantable DC stimulators aiming to deliver tonic DC fields.

### Vascular Changes

Neural tissue is often extensively vascularized due to the high energy requirements of neurons. Externally applied currents can potentially influence ionic movement across the blood-brain barrier via electroporation, electrophoretic or electro-osmotic mechanisms (Bonakdar et al., 2017; Brinton et al., 2018). There has been compelling evidence both *in-vivo* and -*in-vitro* to suggest that both sustained direct current and pulsed electric fields in the brain can transiently modify blood vessel permeability without cell death or electroporation at field strengths relevant to electrical stimulation of neural tissue (Hladovec, 1971; Lopez-Quintero et al., 2010; Brinton et al., 2018). Changes in vascular permeability have been proposed as a possible mechanism contributing to the sustained effects of transcranial DC (Cancel et al., 2018). Locally delivered DC for neural stimulation may output much greater field strengths than in non-invasive DC delivery and proposed uses of invasive DC stimulation involve continuous or near-continuous delivery of DC. Given this, it is important to consider vascular permeability when addressing potential mechanisms for any observed chronic effects of focal DC delivery in the cortex.

### Epithelial Migration and Bone Growth

When the body is injured via physical trauma, a complex host of signaling ques inform surviving tissue to direct cell proliferation and orientation (Singer and Clark, 1999; Gurtner et al., 2008). One of these signaling pathways involves endogenous electric fields, that are thought to direct cells to migrate toward the injured site during wound healing (Jaffe and Vanable, 1984; Zhao, 2009). Endogenous field strengths can be relatively strong, ranging from 40 to 140 mV/mm at the wound edge in epithelial tissue (Jaffe and Vanable, 1984; Chiang et al., 1992); several orders of magnitude larger than in uninjured tissue. These voltages are thought to be generated via ionic currents both as a direct result of the injury (non-ion-specific leakage currents) and as a sustained effect in the surviving tissue, typically via active Na^+^/Cl^−^ transport (Reid et al., 2005; Zhao et al., 2006). The delivery of artificially generated, tonic electric fields at physiologic field strengths could result in an override of endogenous field activity and subsequent cell migration (Zhao et al., 2006).

An invasive device requires a wound for the device to be surgically implanted in the body. Neural implants using DC may also be interfacing with target neural epithelia that has undergone some previous or ongoing degeneration or injury. Given the relative infancy of DC-mediated *in-vivo* neural stimulation there is currently no indication as to the effect of implanted DC stimulation on surrounding tissue response. We might, for example, expect the insertion site of a DC interface to exhibit different tissue responses when it is primarily delivering cathodal vs. anodal constant current. Like with any invasive neural implant, potential tissue reaction will have to be assessed histologically following *in-vivo* implantation and chronic stimulation studies.

Endogenous electric fields also play a similarly important role in bone growth and repair (Fukada and Yasuda, 1957; Konikoff, 1975; McDonald, 1993). Constant current electric stimulation can reliably induce ossification at the cathode, particularly in conjunction with the placement of tissue engineering scaffolds (O'Connor et al., 1969; Buch et al., 1984; Kuzyk and Schemitsch, 2009; Victoria et al., 2009; Griffin and Bayat, 2011; Leppik et al., 2018). DC electric fields are thought to influence ossification primarily via local electrochemical/pH changes at the electrode interface (Brighton et al., 1975; Griffin and Bayat, 2011). Devices that deliver DC for chronic stimulation of neural tissue are unlikely to influence ossification mechanisms as they must mitigate these electrochemical changes in order to function.

## Conclusion

The development of implantable neuromodulation therapies has been historically constrained by the need to deliver short, biphasic pulses to prevent toxic electrochemical reactions forming at the tissue interface. More recent technological advances have enabled the safe delivery of ionic direct current to neural targets. This method of neuromodulation has the potential to significantly diversify our ability to interact with the nervous system by not only being able to excite neural targets, but also being able to suppress neural activity, control AP conduction velocity, and remodel synaptic connections to affect neural processing. Devices that locally deliver DC modulation could greatly expand the range of applications available to neuromodulation therapies beyond what is achievable with standard IPG waveforms.

While pulsatile neuromodulation methods have been thoroughly investigated for safety limitations, the exploration of DC safety is sparse. This is primarily due to the historic inability to deliver DC in an implantable device without violating charge-injection-criteria. As new technology eliminates the problem of metal-tissue stimulation toxicity for ionic DC delivery, new complications will likely be uncovered.

Future applications could use a combination of DC and pulsatile stimuli to even further enhance our ability to modulate the nervous system. Pulsatile delivery methods have an advantage over DC sources in that pulses can be delivered through very small interfaces such as ~10 μm diameter metal electrodes. DC requires larger interfaces (currently the smallest is 200 μm in diameter). One could therefore envision cortical arrays of DC stimulation ports and pulsatile stimulation and recording probes. DC arrays would create localized electric fields that control synaptic connectivity and information flow and implantable recording and stimulation electrodes would take advantage of the modified neural network connections to record from or convey information to the brain in a more controlled fashion.

While DC neuromodulation must address previously un-encountered safety challenges, it offers an exciting possibility of improving machine-neural interfaces in ways that may be difficult or even impossible to achieve otherwise.

## Author Contributions

GF wrote the first half of the manuscript and conducted the compilation and final edits. FA wrote the last half of the manuscript and conducted interim edits of the overall manuscript.

### Conflict of Interest Statement

GF holds the following US patents:

2012 GF, CC Della Santina, “Implantable Vestibular Prosthesis and Methods for Sensing Head Motion and Conveying the Signals Representing Head Movements to the Vestibular Nerve,” JHU US Pat. US20120277835.

2012 GF, CC Della Santina, “Artifact Control and Miniaturization of the Safe DC Stimulator for Neural Prostheses,” JHU US Pat. US20140364796.

GF has submitted the following pending patents for Johns Hopkins internal review:

2016 GF, “Safe Direct Current Stimulation (SDCS) Self-Curling Nerve Cuff Electrode,” JHU C14442.

2017 GF, “Safe Direct Current Stimulator Design for Reduced Power and Increased Reliability,” JHU C14620. The remaining author declares that the research was conducted in the absence of any commercial or financial relationships that could be construed as a potential conflict of interest.
